# Association of Mothers’ Perception of Neighborhood Quality and Maternal Resilience with Risk of Preterm Birth

**DOI:** 10.3390/ijerph120809427

**Published:** 2015-08-12

**Authors:** Namrata Bhatia, Shin Margaret Chao, Chandra Higgins, Suvas Patel, Catherine M. Crespi

**Affiliations:** 1 Department of Epidemiology and Biostatistics, University of California at San Francisco, San Francisco, CA 94158, USA; 2 Los Angeles County, Department of Public Health, Maternal, Child, and Adolescent Health Programs, Los Angeles, CA, USA; E-Mails: schao@ph.lacounty.gov (S.M.C.); chiggins@ph.lacounty.gov (C.H.); supatel@ph.lacounty.gov (S.P.); 3 Department of Biostatistics, University of California at Los Angeles, Los Angeles, CA 94158, USA; E-Mail: ccrespi@g.ucla.edu

**Keywords:** neighborhood quality, maternal resilience, preterm births

## Abstract

We examined the associations of mothers’ perception of neighborhood quality and maternal resilience with risk of preterm birth and whether maternal resilience moderated the effect of neighborhood quality perception. We analyzed data from 10,758 women with singleton births who participated in 2010–2012 Los Angeles Mommy and Baby surveys. Multilevel logistic regression models assessed the effects of mothers’ perception of neighborhood quality and maternal resilience on preterm birth (yes/no), controlling for potential confounders and economic hardship index, a city-level measure of neighborhood quality. Interaction terms were assessed for moderation. Mothers’ perception of neighborhood quality and maternal resilience were each uniquely associated with preterm birth, independent of potential confounders (*p*-values < 0.05). The risk of preterm birth among mothers who perceived their neighborhood as of poor quality was about 30% greater compared to mothers who perceived their neighborhood as of good quality; the risk was 12% greater among mothers with low resilience compared to those with high resilience. Effects of neighborhood quality were not modified by maternal resilience. The findings suggest that mothers’ perception of neighborhood quality and resilience are associated with the risk of preterm birth. Further research should explore whether initiatives aimed at improving neighborhood quality and women’s self-esteem may improve birth outcomes.

## 1. Introduction

Preterm births, defined as births that occurred before 37 weeks of pregnancy, are a serious health concern. Infants born preterm are at a higher risk of early death and lifelong neurologic and cognitive defects than those born later in pregnancy [1]. The prevalence of preterm births in the United States has increased over the past decade, with persistent racial/ethnic disparities [2]. 

A considerable amount of research has been done to identify and understand risk factors for preterm births, including behavioral risk factors and pre-pregnancy health conditions [3,4]. Various stressful events during pregnancy have been shown to have an impact on women’s physical, mental and emotional health and behaviors that may ultimately lead to increased risk of an adverse birth outcome [5]. These findings support that stressors during pregnancy may predispose women to preterm delivery. These stressors may include financial stress, negative life events, catastrophic events and emotional stress [5].

The quality of the neighborhood environment can also be a significant source of stress. There has been increasing investigation on the influence of environment and neighborhood on an individual’s health [4,6,7]. There have been some studies investigating the effects of a socioeconomically-deprived neighborhood on the risk of preterm birth [8]. However, the unique impacts of the quality of a neighborhood on birth outcomes remain poorly understood.

A number of epidemiological studies have investigated the association of mothers’ pre-pregnancy health conditions and psychological state with pregnancy outcomes [3,9]. Support provided by a neighborhood or family, such as providing information or assistance when needed, is thought to influence mothers’ psychosocial processes by providing a support system and enhancement of self-esteem and mutual understanding [6,10]. The association between maternal psychological state during pregnancy and birth outcomes is well established [11,12]. However, there is little literature assessing the association of maternal personal resources, such as self-esteem, self-control and self-efficacy, which we call “maternal resilience,” with preterm birth [9]. Furthermore, there has been little work examining the combined effects of neighborhood and maternal resilience.

In this study, we aimed to investigate whether neighborhood quality and maternal resilience are associated with the risk of preterm birth using a large population-based sample of mothers from Los Angeles County. This research was performed at the Los Angeles County Department of Public Health. Local public health departments may be better equipped and trained to improve maternal resilience, by designing appropriate interventions, compared to neighborhood safety and cleanliness. Hence, hypothesizing that women with high resilience might be better able to overcome the effects of a disadvantaged neighborhood, we also aimed to investigate whether maternal resilience moderates the association of neighborhood quality with preterm birth. 

## 2. Experimental Section

### 2.1. Data Sources

Data for this study were obtained from the 2010 and 2012 Los Angeles Mommy and Baby Surveys (LAMB), combined with information from birth certificates. LAMB is a cross-sectional, population-based survey conducted by the Los Angeles County Department of Public Health (LADPH) every two years since 2005 [13]. The LAMB survey is modeled after the CDC (Center for Disease Control and Prevention) Pregnancy Risk Assessment Monitoring System [13]. This survey collects information from new mothers about events from preconception, prenatal and postpartum experiences. Mothers are surveyed 2–7 months after delivery of the baby. On average, babies are 4 months old at the time of data collection. The majority of mothers completed the survey via mail in English, Spanish or Chinese. Written surveys took approximately 30 minutes. A small percent of surveys were conducted as phone interviews. Phone interviews took about 45 minutes to complete.

LAMB data are used for surveillance and research to monitor, evaluate and improve maternal and child health in Los Angeles County. LAMB employs a stratified random sampling scheme based on Service Planning Areas (SPAs), race/ethnicity and maternal age. African American women and women less than 20 years old were oversampled, and sample weights were assigned to correct for oversampling and allow the representation of population estimates. The response rates for LAMB were 57% in 2010 and 62% in 2012. Birth certificate data of the respondents were linked to their LAMB survey data by LADPH.

The project was approved by the California Committee for the Protection of Human Subjects. Informed consent was obtained from all participants.

### 2.2. Study Sample

Our study sample consisted of 10,758 Los Angeles County resident mothers who gave birth to a live-born singleton in 2010 or 2012 and who participated in the 2010 or 2012 LAMB survey. Respondents who lived in the neighborhood where they spent most part of their pregnancy for less than 1 year and those who were missing information on how long they had lived in the neighborhood were omitted from the analysis. Respondents who were missing information on birth outcome (preterm *vs.* full-term) were also omitted. The study only included singleton births, and hence, twins or other multiples were also excluded. 

### 2.3. Measures

#### 2.3.1. Dependent/Outcome Variable

The outcome for this study was preterm birth (yes/no), defined as a birth that occurred in less than 37 weeks of pregnancy [14]. Information regarding birth outcomes (preterm: yes/no) was obtained from the length of gestation (in days) reported on birth certificates by the doctor based on mother’s last menstrual period. 

#### 2.3.2. Independent Variables

The two main independent variables of interest were neighborhood quality and maternal resilience.

(1) Perceived neighborhood quality: Mothers’ perception about the quality of her neighborhood was assessed using five questions in the LAMB survey that were derived from items used in the Los Angeles Family and Neighborhood Survey (L.A.FANS) [15] and in the Project on Human Development in Chicago Neighborhoods [16]. The questions asked mothers to rate their neighborhood in terms of “police protection”, “protection of property”, “safety from violence”, “cleanliness” and “municipal services” (e.g., trash pickup, road repair, libraries, water). The response format was a Likert scale of 1–5 coding for very poor, poor, neutral, good and very good. Cronbach’s alpha of 0.89 indicated the strong internal consistency of the five items. Scores for the five items were averaged, and a binary perceived neighborhood quality indicator (poor or good) was obtained by classifying mothers with an average score ≤2 (corresponding to the average rating of poor or lower) as perceiving their neighborhood as poor quality and those with an average score >2 as perceiving their neighborhood to be good quality. Neighborhood quality was regarded as missing if responses to any of the five questions were missing.

(2) Maternal resilience: The survey included five questions that asked mothers how strongly they agreed with statements about their self-esteem and perception that they can achieve their goals or complete tasks and control the events affecting them. These questions were based on a modified previously-validated Rosenberg self-esteem scale [17] and Pearlin Mastery scale [18]. The questions asked mothers to rate the following statements with regard to how they felt during their pregnancy: “I feel that I’m a person of worth, at least on an equal plane with others”, “I am able to do things as well as most other people”, “On the whole, I am satisfied with myself”, “I have little control over things that happen to me”, “I can do just about anything I really set my mind to do”. Participants rated these items on a Likert scale of strongly disagree, disagree, neutral, agree and strongly agree, coded as 1–5. Scores for one question were reverse coded to match the direction of the other four questions. Cronbach’s alpha of 0.75 indicated good internal consistency. Scores for the five items were averaged, and a binary maternal resilience indicator (low or high) was obtained by classifying mothers with an average score ≤3 (corresponding to the average rating in the disagree range) as having low resilience and >3 as having high resilience. 

#### 2.3.3. Control Variables

Potential confounders were selected as control variables for the analysis. These covariates included participant sociodemographic characteristics, medical risk factors and health behavior risk factors.

(1) Sociodemographic factors: Sociodemographic factors were maternal race/ethnicity (white, Hispanic, African-American, Asian/Pacific Islander, Native Americans/other/unknown), maternal age (<20 years old, 20–29 years old, 30+ years old), maternal education (less than high school, high school, some college and college graduates), marital status during pregnancy (married, not married) and insurance before pregnancy (yes, no). Sociodemographic information was obtained from the survey data.

(2) Medical risk factors: A pre-pregnancy health conditions indicator variable was coded as “yes” if the respondents reported any of the following conditions before pregnancy in the survey: asthma, hypertension, diabetes, anemia, heart problems or problems with gums or teeth. Otherwise, it was coded as “no”. 

(3) Health behavior risk factors: Adverse health behaviors, such as smoking (primary and passive smoking) and substance use during pregnancy, were also included as potential confounders. Smoking and passive smoking were coded as “yes” if respondents reported smoking or exposure to second-hand smoking during pregnancy in the survey, respectively, and “no” otherwise. Substance use was coded as “yes” if the respondent indicated in the survey using marijuana, amphetamines, cocaine or tranquilizers during pregnancy and “no” otherwise. 

(4) Objective measure of neighborhood quality (neighborhood-level data): The neighborhood-level variable used in this study was the economic hardship index (EHI), developed by the Los Angeles County Department of Public Health. The economic hardship index value is calculated for each city in Los Angeles County with a population larger than 10,000; for the City of Los Angeles, it is calculated for each LA City Council District. The EHI is based on six census tract-level variables: (a) per capital income; (b) percent of persons with less than a high school diploma for a population 25 years and older; (c) percent of persons at less than 200% of the federal poverty level; (d) percent of civilian population over the age of 16 that was unemployed; (e) percent of the population under the age of 18 or over the age of 64; and (f) percent of occupied housing units with more than one person per room. Scores on the index of 1–100 were generated, with a higher score representing a greater level of economic hardship. Details on how the economic hardship index is calculated are available elsewhere [19]. We categorized the index into four categories based on quartiles. A categorical economic hardship index was then used as an objective measure of neighborhood quality.

### 2.4. Statistical Analysis

We summarized the characteristics of our study population using both raw and weighted frequencies, the latter obtained using survey weights. Bivariate associations between the independent variables (neighborhood quality, maternal resilience and covariates) and the dependent variable (preterm birth) were assessed using chi-square tests using the sampling weights. 

We then investigated the association of neighborhood quality and maternal resilience with preterm birth using multilevel logistic regression. The model included the neighborhood quality and maternal resilience indicators and controlled for sociodemographic factors, pre-pregnancy conditions and health behavior risk factors during pregnancy. The model included a random intercept for neighborhood and economic hardship index as the Level 2 (neighborhood level) variable. To investigate the moderation of the effect of neighborhood quality by maternal resilience, we tested an interaction term between these variables. Sampling weights were used in the regression models.

Respondents excluded from the analysis due to missing data on one or more variables were compared to respondents included in the analysis using chi-square tests to assess potential bias due to missing data.

All analyses were conducted in SAS (Version 9.3, SAS Institute, N.C.).

## 3. Results and Discussion

### 3.1. Sample Characteristics

There were a total of 13,436 respondents to the 2010 and 2012 LAMB surveys. After applying the exclusion criteria, 10,758 respondents were included in this study. This corresponded to a weighted frequency of 213,083 mothers in Los Angeles County.

Table 1 shows the characteristics of our study population. Nine percent (n = 950, weighted n = 19,224) of mothers had a preterm birth. Four percent (n = 437, weighted n = 8,320) of mothers perceived their neighborhood as poor quality, and eight percent (n = 811, weighted n = 15,821) of mothers perceived themselves to have low resilience. About half of the mothers (53%) in our study attended some college or had a college degree. Over half (60%) of the mothers were Hispanic; 18% were white; 13% were Asian/Pacific Islander; and 7% were African Americans. Half of the respondents were over 30 years of age; over half were married (57%); and about 34% did not have health insurance. A small percentage of mothers (2%) smoked during pregnancy, and 10% were exposed to second-hand smoking during pregnancy. Over a quarter of the mothers (27%) had pre-pregnancy conditions.

**Table 1:** Characteristics of the study population.

Variables	Frequency	Weighted Frequency	%	SE of %
Preterm birth				
No	9,808	193,860	90.98	0.43
Yes	950	19,224	9.02	0.43
Missing	0			
Maternal education				
Less than high school	1,876	48,419	23.30	0.67
High school graduate	2,250	49,093	23.63	0.64
Some college	6,049	110,288	53.07	0.76
Missing	583			
Mother’s race/ethnic group				
White	2,639	37,925	17.80	0.43
Hispanic	3,679	129,128	60.60	0.69
Black	1,765	15,689	7.36	0.22
Asian/Pacific Islander	1,979	27,185	12.76	0.58
Native American/other/unknown	696	3,156	1.48	0.07
Missing	0			
Mother’s age				
<20 years old	1,900	15,756	7.39	0.21
20–29 years old	3,626	90,651	42.54	0.74
30+ years old	5,232	106,676	50.06	0.75
Missing	0			
Marital status				
Married	5,892	119,960	56.94	0.74
Not married	4,742	90,732	43.06	0.74
Missing	124			
Insurance status				
No insurance	2,701	71,954	33.89	0.78
Had insurance	8,025	140,337	66.11	0.78
Missing	32			
Smoking during pregnancy				
No	10,243	204,894	97.81	0.18
Yes	336	4,589	2.19	0.18
Missing	179			
Passive smoking during pregnancy				
No	9,692	192,387	90.29	0.43
Yes	1,066	20,696	9.71	0.43
Missing	0			
Substance abuse during pregnancy				
No	10,313	203,960	98.30	0.17
Yes	228	3,524	1.70	0.17
Missing	217			
Pre-pregnancy conditions during pregnancy				
No	7,846	156,007	73.21	0.64
Yes	2,912	57,076	26.79	0.64
Missing	0			
Mothers’ perception of neighborhood quality				
Good quality	10,184	200,585	96.02	0.27
Poor quality	437	8,320	3.98	0.27
Missing	137			
Maternal resilience				
High maternal resilience	9,748	191,790	92.38	0.38
Low maternal resilience	811	15,821	7.62	0.38
Missing	199			
* Original N = 13,436; after exclusion criteria: final N = 10,758 (weighted N = 213,083)				

### 3.2. Bivariate Analysis

Bivariate analysis (Table 2) indicated that maternal race/ethnic group, age, education, insurance status, marital status, pre-pregnancy conditions, smoking and substance abuse during pregnancy were each associated with preterm birth outcomes. Mothers who perceived their neighborhood as poor quality had significantly more preterm birth outcomes (10.4%) compared to mothers who perceived their neighborhood as good quality (8.8%). Mothers who perceived themselves to have low resilience had significantly more preterm birth outcomes (10.3%) compared to mothers perceiving themselves to have high resilience (8.7%). 

**Table 2:** Prevalence of preterm births by selected characteristics.

Independent Variables	Preterm % N (%) Weighed N		Chi-Square Statistic	*p*-value
Maternal education				
Less than high school	192 (9.7)	4700		
High school graduate	199 (8.9)	4349		
Some college	505 (8.8)	9674	38.9	<0.01
Mother’s race/ethnic group				
White	176 (6.9)	2629		
Hispanic	347 (9.4)	12,133		
Black	209 (12.7)	1986		
Asian/Pacific Islander	157 (8.1)	2184		
Native American/other/unknown	61 (9.3)	292	509.5	<0.01
Mother’s age				
<20 years old	195 (11.4)	1788		
20–29 years old	292 (8.2)	7450		
30+ years old	463 (9.4)	9986	189.9	<0.01
Marital status				
Married	434 (8.0)	9621		
Not married	500 (10.2)	9210	445.9	<0.01
Pre-pregnancy insurance status				
No insurance	233 (9.3)	6686		
Had insurance	717 (8.9)	12,538	86.3	<0.01
Smoking during pregnancy				
No	882 (8.9)	18,315		
Yes	46 (10.2)	466	55.5	<0.01
Passive smoking during pregnancy				
No	839 (9.0)	17,359		
Yes	111 (9.0)	1864	0.0	0.93
Substance abuse during pregnancy				
No	908 (8.9)	18,237		
Yes	26, 16.0	564	226.3	<0.01
Pre-pregnancy conditions during pregnancy				
No	629 (8.6)	13,458		
Yes	321 (10.1)	5765	110.7	<0.01
Mothers’ perception of neighborhood quality				
Good quality	715 (8.8)	14,717		
Poor quality	211 (10.4)	3465	242.3	<0.01
Maternal resilience				
High maternal resilience	837 (8.7)	16,397		
Low maternal resilience	86 (10.3)	1764	612.4	<0.01

### 3.3. Multilevel Logistic Regression Modeling Results

Table 3 provides results for the multilevel model assessing the association of preterm birth with mother’s perception of her neighborhood quality and her resilience controlling for sociodemographic factors, medical risk factors and adverse health behaviors, including the economic hardship index as a Level 2 (neighborhood) variable. Both mothers’ perception of neighborhood quality and maternal resilience were independently associated with the risk of preterm birth (*p*-values < 0.01). The interaction term between these two variables was not statistically significant (*p*-value = 0.4) and, hence, was dropped from the model. According to the final model, controlling for all potential confounders, mothers who perceived their neighborhood as that of poor quality had 1.3-times higher risk (95% confidence interval: [1.2, 1.4]) of having a preterm birth outcome compared to those who perceived their neighborhood as that of good quality. Mothers with low resilience had 1.12-times higher risk (95% confidence interval: [1.05, 1.19]) of having a preterm birth outcome compared to those with high resilience.

African-American, Hispanic, Asian/Pacific Islander and Native American women had higher risk of preterm birth compared to their white counterparts. Unmarried women also had a higher risk of preterm births compared to their married counterparts. Smoking and substance use during pregnancy were associated with increased risk of preterm birth. Unexpectedly, secondhand smoke exposure was associated with lower risk of preterm birth, controlling for other variables. The variance of the random intercept was non-zero (variance of random intercept = 0.83, *p*-value <0.05), implying that there was additional variation in preterm outcomes at the neighborhood level that was not explained by the economic hardship index.

**Table 3:** Multi-level model results for the association of preterm birth with mothers’ perception of neighborhood quality and maternal resilience, controlling for sociodemographic factors, pre-pregnancy conditions, adverse health behaviors during pregnancy and the economic hardship index as a neighborhood-level variable.

Variables	OR	95% CI		*p*-value
Mother’s race/ethnic group				
Asian/Pacific Islander	1.27	1.19	1.36	<0.01
African-American	1.79	1.65	1.93	<0.01
Hispanic	1.26	1.19	1.34	<0.01
Native American/other/unknown	1.54	1.25	1.90	<0.01
White (Ref.)	1.00			
Mother’s age				
20–29 years old	0.77	0.72	0.83	<0.01
30+ years old	1.05	0.98	1.12	0.20
<20 years old (Ref.)	1.00			
Maternal education				
High school graduate	0.84	0.80	0.88	<0.01
Less than high school	0.96	0.91	1.01	0.12
Some college (Ref.)	1.00			
Marital status				
Not married	1.18	1.13	1.23	<0.01
Married (Ref.)	1.00			
Pre-pregnancy insurance status				
Not insured	1.00	0.96	1.04	0.95
Insured (Ref.)	1.00			
Smoking during pregnancy				
Smoker during pregnancy	1.17	1.05	1.30	0.01
Never smoked during pregnancy (Ref.)	1.00			
Passive smoking during pregnancy				
Second-hand smoker during pregnancy	0.89	0.84	0.95	<0.01
Not exposed to second-hand smoking during pregnancy (Ref.)	1.00			
Substance abuse during pregnancy				
Substance abuse during pregnancy	1.64	1.46	1.85	<0.01
No substance abuse during pregnancy (Ref.)	1.00			
Pre-pregnancy conditions during pregnancy				
Pre-pregnancy conditions	1.03	0.99	1.07	0.14
No pre-pregnancy conditions (Ref.)	1.00			
Economic Hardship Index (EHI)				
EHI Quartile 2	2.25	1.36	3.75	<0.01
EHI Quartile 3	2.16	1.19	3.90	0.01
EHI Quartile 4	2.59	1.54	4.36	<0.01
EHI Quartile 1 (Ref.)	1.00			
Mothers’ perception of neighborhood quality				
Poor perceived neighborhood quality	1.30	1.20	1.41	<0.01
Good perceived neighborhood quality (Ref.)	1.00			
Maternal resilience				
Low maternal resilience	1.12	1.05	1.19	<0.01
High maternal resilience (Ref.)	1.00			

### 3.4. Comparison of Included and Excluded Participants

Overall, 21% respondents (n = 2289 out of n = 10,758) were excluded from the analytic dataset due to missing data. About 50% of the excluded respondents (n = 1152 out of 2289) were missing information on the economic hardship index due to sampling from unincorporated communities in Los Angeles County. Chi-square tests comparing included and excluded participants on maternal race/ethnic group, education, marital status, insurance status, passive smoking and substance abuse were statistically significant. However, chi-square tests on the key variables of interest, preterm birth, neighborhood quality and maternal resilience, were non-significant (*p*-values >0.05). Results of these comparisons are summarized in Table 4.

**Table 4:** Comparison of included and excluded survey respondents.

Variables	Excluded Participants N = 2289	Included Participants N = 8469	Chi-Square *p*-value
	Frequency (%)	Frequency (%)	
Preterm births			
No	2,081 (90.6)	7,727 (91.1)	
Yes	208 (9.4)	742 (8.9)	
Missing	0		0.33
Maternal education			
<HS	428 (30.0)	1,448 (21.6)	
HS	458 (26.1)	1,792 (23.0)	
Some college and college grad +	820 (43.8)	5,229 (55.4)	
Missing	583		<0.01
Mother’s race/ethnic group			
White	394 (11.4)	2,245 (19.6)	
Hispanic	830 (65.8)	2,849 (59.1)	
Black	333 (6.2)	1,432 (7.7)	
Asian/Pacific Islander	274 (11.9)	1,705 (13.0)	
Native American/other/unknown	458 (4.7)	238 (0.6)	
Missing	0		<0.01
Mother’s age			
<20 years old	410 (7.8)	1,490 (7.3)	
20–29 years old	815 (43.2)	2,811 (42.3)	
30+ years old	1,064 (48.9)	4,168 (50.4)	
Missing	0		0.05
Marital status			
Married	1,147 (53.4)	4,745 (57.9)	
Not married	1,018 (46.7)	3,724 (42.1)	
Missing	124		0.01
Insurance status			
No insurance	619 (38.6)	2,082 (32.6)	
Had insurance	1,638 (61.4)	6,387 (67.4)	
Missing	32		0.01
Smoking during pregnancy			
No	2040 (97.7)	8,203 (97.8)	
Yes	70 (2.3)	266 (2.2)	
Missing	179		0.68
Passive smoking during pregnancy			
No	1,979 (86.4)	7,713 (91.8)	
Yes	310 (13.6)	756 (8.6)	
Missing	0		<0.01
Substance abuse during pregnancy			
No	2,009 (97.5)	8,304 (98.5)	
Yes	63 (2.5)	165 (1.5)	
Missing	217		<0.01
Pre-pregnancy conditions			
No	1,652 (71.2)	6,194 (73.8)	
Yes	637 (28.8)	2275 (26.2)	
Missing	0		0.35
Economic Hardship Index (EHI)			
EHI Quartile 1	241 (18.2)	2,203 (20.2)	
EHI Quartile 2	328 (23.3)	2,049 (26.3)	
EHI Quartile 3	255 (21.3)	2,062 (20.2)	
EHI Quartile 4	313 (37.2)	2,155 (33.3)	
Missing	1152		<0.01
Mothers’ perception of neighborhood Quality			
Good quality	2,061 (95.4)	8,123 (96.2)	
Poor quality	91 (4.5)	346 (3.84)	
Missing	137		0.76
Maternal resilience			
High maternal resilience	1,921 (91.9)	7,827 (92.5)	
Low maternal resilience	169 (8.1)	642 (7.5)	
Missing	199		0.44

## 4. Discussion

Stress is known to be a risk factor for preterm birth [5]. There are numerous factors that can result in stress on the mother during pregnancy, and neighborhood quality is one of them. Previous research has shown adverse effects of socioeconomically-deprived neighborhoods on birth outcomes [8]. Numerous studies have also shown positive impacts of access to resources on health in general and the impact of mothers’ psychological state during pregnancy on pregnancy outcomes. Most studies have looked at the effects of neighborhood quality and mothers’ psychological state on pregnancy outcomes separately [4,6,11]. In our study, we investigated the combined effects of mothers’ perception of neighborhood quality and maternal resilience on preterm birth outcomes. Neighborhood quality was defined by mothers’ perception of her neighborhood quality in terms of neighborhood safety and cleanliness. Maternal resilience was defined by mothers’ perception of her self-esteem and self-efficacy, and low maternal resilience signified personal dissatisfaction, lower self-worth and sense of self-efficacy, which could negatively affect a mother’s response to an adversity during pregnancy. We also controlled for an objective measure of neighborhood quality, the economic hardship index, in order to enable us to assess the effect of mothers’ perception of neighborhood quality for mothers in neighborhoods with similar socioeconomic characteristics.

Our results suggested that controlling for potential confounders and neighborhood socioeconomic characteristics, perceived neighborhood quality and maternal resilience were each significantly associated with preterm birth outcomes. All else equal, the risk of having a preterm birth outcome among mothers who perceived of their neighborhood as that of poor quality were about 30% greater compared to mothers who perceived of their neighborhood as that of good quality; and the risk of having a preterm birth outcome among mothers with low resilience was about 12% greater compared to mothers with high resilience. However, we did not find evidence that the effect of perceived poor quality neighborhood differed depending on a mother’s resilience.

The economic hardship index used in our study was similar to the census variable-based neighborhood deprivation measure used by O’Campo *et al.* [8]. Studying non-Hispanic black and white women, these authors found that higher neighborhood deprivation was associated with increased risk of preterm birth in both groups. Our study contributes to the literature by finding a further independent association of preterm birth outcomes with self-perceived neighborhood quality, over and above the association with objectively-measured neighborhood socioeconomic characteristics. Our neighborhood quality indicator captured perceived safety and cleanliness of the neighborhood, characteristics that are distinct from the socioeconomic measures. Our findings suggest that concerns about safety, cleanliness and poor municipal services in their neighborhoods may represent additional stressors for pregnant women that can increase their risk for adverse birth outcomes. 

Our findings also resonate with previous studies on the association between mothers’ psychological state and personal resources with birth outcomes [9,11,12], with more resilient women having a lower risk of preterm birth. Our study also found significant associations of maternal race/ethnic group and marital status with risk of preterm birth. Smoking and substance use during pregnancy were associated with increased risk of preterm births. Unexpectedly, secondhand smoke exposure was associated with a lower risk of preterm births in the multivariable analysis. This result could be attributable to incomplete control for confounding or exposure misclassification due to reporting bias.

Our study has several limitations. Women were asked after birth about their experiences during pregnancy, and reporting bias, recall bias and social desirability bias may have influenced their responses. Further, their birth outcome may have influenced their responses, such that they were more likely to provide negative responses about resilience and neighborhood if they had experienced a preterm birth. Thus, while this study provides important suggestions for future research and program direction, causality or even directionality cannot be inferred.

Second, we were missing the economic hardship index for 1152 respondents. The economic hardship index was only calculated for cities in Los Angeles County that had a population larger than 10,000 and was not calculated for unincorporated communities of Los Angeles County. Thus, the LAMB survey respondents from unincorporated communities were excluded from the analysis. The lack of significant difference in preterm birth outcomes, mothers’ perception of neighborhood quality and maternal resilience between those included and excluded in analyses suggested that excluded respondents were comparable to included respondents on the main characteristics of interest. However, the generalizability of our results may be limited to these relatively larger cities in Los Angeles County, and applying our results to populations living in other geographical areas should be done with caution. We used an indicator of objective neighborhood quality at the city and council district level, which may not have well represented the local neighborhoods of the participants. Other types of geographic areas, such as census tract, could be explored in the future.

The neighborhood quality scale was derived from items used in the Los Angeles Family and Neighborhood Survey (L.A.FANS) [15] and in the Project on Human Development in Chicago Neighborhoods [16]. This scale has been used to assess neighborhood quality and has been found to correlate well with other measures of neighborhood characteristics and health outcomes in the L.A.FANS [20,21,22]. However, we did not specifically validate the neighborhood quality scale in our population. The maternal resilience items were derived from the Rosenberg self-esteem scale [15] and the Pearlin Mastery scale [16]. While some work has been done to validate the Rosenberg self-esteem scale (see, for example, [23]), the Pearlin Mastery scale has been widely used in multiple populations, and we did not specifically validate the resilience scale in our population. 

Our study has several strengths. We had a large and population-based sample for this study, and we also systematically controlled for many potential confounders in our statistical models. However, we cannot rule out the possibility of unmeasured confounding. 

In Los Angeles County, about 10% of births, corresponding to about 10,000 births each year, are preterm. Infants born preterm are at higher lifelong risks of neurologic and cognitive defects [1]. Moreover, the costs associated with preterm births to healthcare are enormous. Our study suggests the importance of maternal empowerment and improving the quality of neighborhoods in Los Angeles County to improve birth outcomes. This research provides further support for current and future programs that empower women to improve their personal satisfaction, self-worth and sense of self-efficacy. In particular, local public health departments planning interventions for women predisposed to poor birth outcomes may consider including programming to enhance these aspects of maternal resilience. This research also provides support for a connection between neighborhood safety and cleanliness and health outcomes, which could potentially be addressed through neighborhood programs and initiatives.

Our study supports the premise that interventions designed to increase maternal resilience and to improve neighborhood quality in terms of safety and cleanliness may be beneficial to improve birth outcomes. Though it is difficult to change neighborhood quality dramatically without policy implementations, interventions to increase maternal resilience may be more easily designed and implemented and may have other beneficial consequences. 

## 5. Conclusions

Our results suggested that controlling for potential confounders and neighborhood socioeconomic characteristics, perceived neighborhood quality and maternal resilience were each significantly associated with the risk of preterm birth. We did not find evidence of maternal resilience moderating the association of neighborhood quality and risk of preterm birth. To help us design more effective interventions, further studies are needed to investigate the relationships among neighborhood quality, maternal resilience and preterm births and to elucidate the underlying mechanisms.
